# Extensive reproductive disruption, ovarian masculinization and aromatase suppression in Atlantic croaker in the northern Gulf of Mexico hypoxic zone

**DOI:** 10.1098/rspb.2011.0529

**Published:** 2011-05-25

**Authors:** Peter Thomas, Md. Saydur Rahman

**Affiliations:** The University of Texas at Austin, Marine Science Institute, 750 Channel View Drive, Port Aransas, TX 78373, USA

**Keywords:** northern Gulf of Mexico, hypoxia, reproductive impairment, ovarian masculinization, endocrine disruption, Atlantic croaker

## Abstract

The long-term impacts on marine ecosystems of the recent dramatic worldwide increase in the incidence of coastal hypoxia are unknown. Here, we show widespread reproductive disruption in Atlantic croakers collected from hypoxic sites approximately 120 km apart in the extensive northern Gulf of Mexico continental shelf hypoxic zone. Gonadal growth and gamete production were impaired in croakers from hypoxic sites compared with fish from reference normoxic sites east of the Mississippi River Delta. Male germ cells were detected in approximately 19 per cent of croaker ovaries collected in the hypoxic region, but were absent in ovaries from normoxic sites. In addition, the sex ratio was skewed towards males at the hypoxic sites. The masculinization and other reproductive disruptions were associated with declines in neuroendocrine function, as well as ovarian and brain expression of aromatase (the enzyme that converts androgens to oestrogens). A similar incidence of ovarian masculinization and decline in ovarian aromatase expression were observed in croaker after chronic laboratory hypoxia exposure, indicating that ovarian masculinization is a specific hypoxia response and is due to decreased aromatase activity. The results suggest severe reproductive impairment can occur over large coastal regions in marine fish populations exposed to seasonal hypoxia, with potential long-term impacts on population abundance.

## 1. Introduction

One of the most dramatic global changes owing to human activities over the last half-century has been the marked increase in the incidence and the duration of seasonal hypoxia in estuarine and coastal marine environments. Over 400 coastal hypoxic zones (dissolved oxygen, DO: less than 2 mg l^−1^) covering a total area of approximately 250 000 km^2^ have been identified throughout the world, many of them in regions that until recently had normal DO levels (normoxic, approx. 7 mg l^−1^) in their bottom waters [1]. This spread in coastal hypoxia is primarily owing to eutrophication caused by increased anthropogenic inputs of nitrogen and phosphorus into coastal watersheds, resulting in greater amounts of organic material sinking into bottom waters where increased aerobic microbial decomposition leads to depletion of DO [2]. It is predicted that the extent of coastal seasonal hypoxia throughout the world will continue to increase with the growth of human coastal populations and expansion of intense agricultural practices, and potentially also with global warming [1,3]. Therefore, it is important to determine the long-term impacts of recurring seasonal hypoxia on the resilience of coastal marine ecosystems and the maintenance of fishery resources [4,5]. However, there have been relatively few studies on the chronic effects of sublethal hypoxia stress on processes in marine organisms that affect population abundance, such as reproduction [6,7].

Reproduction is one of the most critical life-history stages that is disrupted by environmental stressors because even minor persistent declines in gamete production (fecundity) by individuals can have long-term impacts at higher levels of biological organization, leading to population declines and community disturbance [8]. The timing and the regulation of gonadal differentiation, the reproductive cycle and gamete production in fish are under precise endocrine control by a suite of hormones secreted by the hypothalamus–pituitary–gonadal axis [9]. Gonadal differentiation and gamete production are especially susceptible to interference by a wide range of sublethal chemical and physical environmental stressors that affect reproductive endocrine function [9,10]. Recent laboratory experiments and two field studies have shown marked impairment of reproduction and endocrine function in the Atlantic croaker (*Micropogonias undulatus*) and several other teleost species chronically exposed to hypoxia [7,11–13]. Gametogenesis and endocrine function were impaired in both male and female croakers chronically exposed to hypoxia in East Bay, a Florida estuary, whereas croakers in the adjoining normoxic Pensacola Bay showed normal reproductive development and endocrine signalling [7]. The patterns of reproductive and endocrine impairment in croakers from East Bay were very similar to those in croakers in the laboratory hypoxia study, suggesting that these responses are highly specific to hypoxia. However, it is not known whether environmental hypoxia in more open coastal marine environments (where fish can readily avoid low DO areas) causes reproductive impairment, nor whether it occurs over the larger spatial scales in these regions, where hypoxia has the potential to have much greater ecological and population impacts.

The aim of the present study was to determine whether reproductive and endocrine impairment occurs in croakers collected over a broad region in the northern Gulf of Mexico (nGOM) continental shelf hypoxic zone, the second largest seasonal hypoxic zone in the world. Six hypoxic sites were sampled along two transects approximately 120 km apart (which included most of the eastern half of the hypoxic zone off the Louisiana coast) and three reference sites were sampled east of the Mississippi River Delta (which are usually normoxic during the summer months) [14,15]. A long-term goal of this research is to provide data that can be used to predict the population impacts of persistent recurring seasonal hypoxia in the nGOM.

## 2. Material and methods

### (a) Description of field sites

The nGOM off the Louisiana coastline experiences widespread seasonal hypoxia each year from late May until the end of September, with considerable interannual temporal and spatial variation [14]. Hypoxia occurs in shallow (5–60 m) bottom waters, but is most common in the depth range of 5–30 m [14]. The extent of seasonal hypoxia in the nGOM has increased from an average area of 8300 km^2^ during the late 1980s to over 16 000 km^2^ in 1990s, and has averaged 16 700 km^2^ since then [14,15]. Hypoxic sites (20–33 m deep) were selected along two transects (three per transect) that had been regularly monitored for bottom DO on a monthly (transect C) or bimonthly (transect F) basis over several years and were found to be frequently hypoxic during the summer months [14]. Twenty years of monitoring data for transect C off Terrebonne Bay show that hypoxia occurs as early as late February and lasts until early October, with continuous low DO typically observed from the middle of May until the middle of September [14]. The more limited monitoring data for transect F off Atchafalaya Bay collected from 2001 onwards show hypoxia typically develops later in the spring and ceases one month earlier than at transect C, and is also less persistent and frequently moderately hypoxic (DO: 2–3.5 mg l^−1^) [14]. These two areas of the Louisiana coastal region are influenced by different riverine inputs—the Mississippi River (transect C) and Atachafalaya River (transect F)—and comprise distinct hypoxic regions [15]. Reference sites less than 30 m deep and approximately 180 km from the nearest hypoxic zone site, which are usually normoxic, were selected east of the Mississippi Delta because hypoxia there is sporadic, covers a limited area and is short-lived (electronic supplementary material, figure S1) [14,15].

### (b) Collection of field samples

Twenty adult young-of-the-year (yr1) Atlantic croakers (both sexes; mean length: 15.7 cm) were collected with 15 min bottom trawls from the RV *Pelican* from 27 September to 2 October 2007 from the three normoxic sites east of the Mississippi River Delta and the six sites in the hypoxic zone along the C and F transects (electronic supplementary material, figure S1). Fish were held in running sea water 5–10 min after capture, prior to blood collection from the caudal vein with a heparinized syringe containing aprotonin (Sigma, St. Louis, MO, USA), and removal of brain, liver and gonadal tissues. Plasma and tissues were rapidly frozen in liquid nitrogen, shipped to the laboratory, and stored at −80°C until analysed. Another 30–40 fish (size range: 15–18 cm) were collected at each sampling site and their gonads were examined to estimate the sex ratio. The sex ratio was also determined for croakers collected during October 2007 by Louisiana Department of Wildlife and Fisheries (LDWF) and during October–November 2007 by National Oceanic and Atmospheric Administration (NOAA).

### (c) Laboratory hypoxia experiments

For one month prior to experimentation, approximately seven-month-old croakers (mean length: 10.7 cm, body weight, BW: 13.7 g) obtained from local fishermen in May 2007 were acclimated to laboratory conditions in recirculating sea water tanks at the University of Texas Marine Science Institute (UTMSI) and fed chopped shrimp (3.5% BW d^−1^). Fish were transferred in June into nine 1650 l recirculating tanks (30 fish per tank) and continuously exposed to normoxia (DO: 6.5 mg l^−1^; three tanks) or hypoxia (DO: 2.0 mg l^−1^; six tanks) conditions for 15 weeks. After the 15-week exposure period, the DO in three hypoxic tanks was adjusted to 2.7 mg l^−1^ (moderate hypoxia) to maintain food consumption, while the DO in the remainder was adjusted to 6.5 mg l^−1^ (normoxia) and the exposure continued for an additional five weeks. The DO levels in the hypoxia exposure tanks were lowered by reducing the aeration gradually, as described previously (electronic supplementary material, figure S2) [7]. At the end of the experiments, the fish were sacrificed under deep anaesthesia using quinaldine sulphate (20 mg l^−1^), following guidelines approved by the University of Texas at Austin Animal Care and Use Committee. Tissues were rapidly excised, frozen in liquid nitrogen and stored at −80°C until analysed.

### (d) Gonadosomatic index and gonadal histology

Total length, BW and gonad weight (GW) were measured, and gonadosomatic index (GSI) was calculated from the formula GW(BW − GW)^−1^ × 100. The proportion of oocytes at each developmental stage was determined in fixed ovarian sections, as described previously [7]. Criteria for positive identification of male germ cells in ovaries included clear identification of several spermatogenic stages and/or the diameter of spermatogenic areas greater than or equal to 10 µm.

### (e) Fecundity

Fecundity was calculated from estimates of the number of fully grown vitellogenic oocytes (diameter: greater than 350 µm), obtained by counting the oocytes in aliquots of dissociated ovarian tissue, as described previously [16].

### (f) Plasma vitellogenin and testosterone

Plasma vitellogenin (VTG) and testosterone (T) concentrations were measured by sandwich enzyme-linked immunosorbant assay (ELISA) and radioimmunoassay, respectively [7].

### (g) Quantification of oestrogen receptor alpha, gonadotrophin-releasing hormone-I, gonadal aromatase and brain aromatase mRNA levels

Total RNA was extracted from liver, ovary and brain tissues using TRI reagent (Sigma) and treated with DNase I (Promega, Madison, WI, USA) according to the manufacturer's instructions. Gene-specific primers for oestrogen receptor alpha (*er*α**), gonadotrophin-releasing hormone-I (*gnrh-I*) and gonadal aromatase (*cyp19a1a*) and brain aromatase (*cyp19a1b*) were designed from the nucleotide sequences of croaker *er*α**, *gnrh-I*, *cyp19a1a* and *cyp19a1b* (electronic supplementary material, table S1). Croaker *18S* rRNA was used as an internal control. Quantitative real-time PCR (qRT-PCR) was conducted to determine the relative gene expression. The analysis of relative mRNA expression results was performed using the 2^−*ΔΔ*C^^*t*^ method [17].

### (h) Aromatase activity

Aromatase activity in the ovary was measured by a radioenzymatic assay as described previously [18].

#### (i) Statistical analysis

Significant differences between field data for normoxic and hypoxic sites were analysed by nested ANOVA, and laboratory data by one-way ANOVA. Where significant interactions were found, this was followed by Fisher's protected least-significant difference (PLSD) test for multiple comparisons and Student's *t*-test for paired comparisons. For the sex ratio estimation, UTMSI and NOAA data were analysed by nested ANOVA, and LDWF data were analysed by a binomial test.

## 3. Results

### (a) Dissolved oxygen values at the sampling sites

The bottom waters at sites C5, C6 and C7 had become hypoxic by May 2007, whereas on transect F only site F3 was hypoxic at this time (F4 was moderately hypoxic), similar to the findings in previous years (electronic supplementary material, table S2) [14]. In July, all the C sites and F3 site were hypoxic, and F4 and F5 sites were moderately hypoxic (electronic supplementary material, table S3). Similarly, during our 11–13 August cruise, the F4 and F5 sites were moderately hypoxic (DO 2.4 and 3.2 mg l^−1^, respectively; electronic supplementary material, table S4). During the 20 August cruise (electronic supplementary material, table S2), all the sites on transect C and F3 and F5 were hypoxic (F4 was not monitored). On 12 September, the bottom DO at C5, C6 and C7 was less than 1.0 mg l^−1^, whereas sites F3, F4 and F5 had become moderately hypoxic to normoxic (DO: 6.1, 2.3 and 4.5 mg l^−1^ respectively; electronic supplementary material, table S3). Thus, the data suggest that the three sites along the C transect remained hypoxic throughout the summer until mid-September, whereas the DO in the bottom waters at the F transect sites fluctuated and hypoxia was intermittent. In contrast, all three reference sites east of the Mississippi River Delta were normoxic in August, when the extent of the hypoxia region along the western Louisiana coast is maximal (electronic supplementary material, figure S1 and table S4). A similar summer pattern of normoxic bottom water (DO: 4.3–4.6 mg l^−1^) was seen at the reference sites in July 2008 [19].

### (b) Ovarian growth and oogenesis

**Figure 1.**
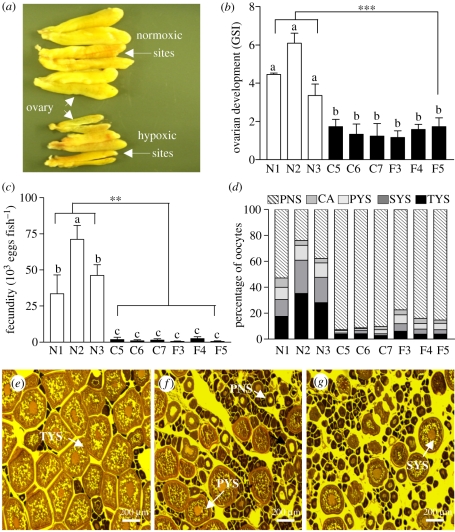
(*a*,*b*) Ovarian growth, (*c*) fecundity and (*d*–*g*) oogenesis in female croakers collected from six hypoxic sites (C5–C7, F3–F5) and three normoxic sites (N1–N3) in the nGOM during 27 September–2 October 2007. (*a*) Gross appearance of representative ovaries from hypoxic and normoxic sites. (*b*) Gonadosomatic index (GSI), an index of ovarian growth. (*c*) Fecundity. (*d*) Percentage of oocytes at each development stage. PNS, peri-nucleolus; CA, cortical alveoli; PYS, primary yolk; SYS, secondary yolk; TYS, tertiary yolk stage. (*e–g*) Histological appearance of representative ovaries collected from the (*e*) reference and (*f*) C transect and (*g*) F transect hypoxic zone sites. For site locations and water quality parameters see electronic supplementary material, figure S1 and tables S2–S4. Asterisks denote significant differences between normoxic (reference) and hypoxic sites (nested ANOVA, **p < 0.01, ***p < 0.001). Each value represents the mean ± s.e.m. (*n* = 8–12). Individual site differences are indicated with different letters (Fisher's PLSD, *p* < 0.05).

Gross examination indicated that the ovaries of croakers collected from the hypoxic sites were at an early stage of vitellogenic growth, whereas the ovaries collected from the reference sites were at a medium to advanced stage of ovarian growth (figure 1*a*). The GSI of females collected from the six hypoxic sites on transects C and F were similar (mean 1.46 ± 0.1), and less than 50 per cent of those at the three reference sites (mean 4.64 ± 0.79, *p* < 0.001; figure 1*b*). There was a dramatic reduction in the production of fully grown oocytes (oocyte diameter: greater than 350 µm) at the hypoxic sites (mean: less than 1300 eggs fish^−1^) compared with the reference sites (7700–124 600 eggs fish^−1^; figure 1*c*). The ovaries were at early stages of oogenesis in fish from the hypoxic sites and contained relatively few fully grown tertiary yolk stage vitellogenic oocytes, whereas the ovaries obtained at the reference sites were full of large tertiary yolk stage oocytes (figure 1*e–g*). Histological examination of ovarian sections revealed that 80 to 90 per cent of the oocytes were arrested at the peri-nucleolar stage in fish at the hypoxic sites, and only 10 to 20 per cent were at primary, secondary or tertiary yolk stages (figure 1*d*). By contrast, 40 to 70 per cent of the oocytes were at various stages of vitellogenic growth in fish from the reference sites, and 18 to 35 per cent had reached the tertiary yolk stage.

### (c) Testicular growth and spermatogenesis

**Figure 2.**
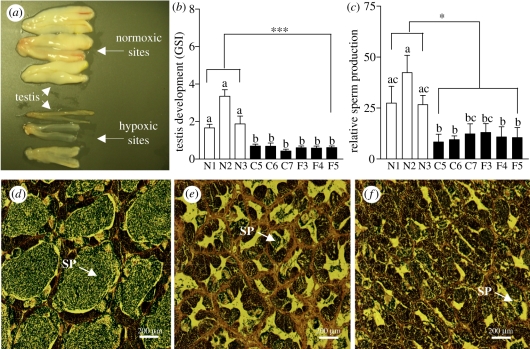
(*a,b*) Testicular growth, (*c*) sperm production and (*d,e,f*) spermatogenesis in male Atlantic croakers collected from six sites in the hypoxic coastal region (C5–C7, F3–F5) in the nGOM and from three reference normoxic sites (N1–N3) during 27 September–2 October 2007. (*a*) Gross appearance of representative testes from hypoxic and normoxic sites. (*b*) Gonadosomatic index (GSI). (*c*) Sperm production. (*d*–*f*) Histological appearance of representative testes collected from the (*d*) reference and (*e*) C transect and (*f*) F transect hypoxic zone sites. SP, spermatozoa. Asterisks denote significant differences between normoxic (reference) and hypoxic sites (nested ANOVA, ***p < 0.001, *p < 0.05). Each value represents the mean ± s.e.m. (*n* = 6−16). Individual site differences are indicated with different letters (Fisher's PLSD, *p* < 0.05).

There was a marked impairment of testicular growth and spermatogenesis in croakers at the hypoxic sites compared with that observed at the normoxic sites (figure 2*a–f*). No milt was released from males upon applying gentle pressure to the abdomen, whereas milt flowed freely from most of the males at the reference sites. The mean GSI of males from the hypoxic sites were uniformly low (mean 0.6 ± 0.04), less than half of those at the three reference sites (mean 2.29 ± 0.53; figure 2*b*). Relative sperm production was also significantly lower (figure 2*c*). The tubules of testes collected at the reference sites had large lumens (mean diameter: 406 ± 18 µm), full of spermatozoa, which were surrounded by a single layer of cysts containing earlier spermatogenic stages (figure 2*d*), whereas the testicular tubules of males from the hypoxic sites had much smaller lumens (mean diameter: 185 ± 19 µm) containing only a few spermatozoa (figure 2*e,f*).

### (d) Ovarian masculinization

**Figure 3.**
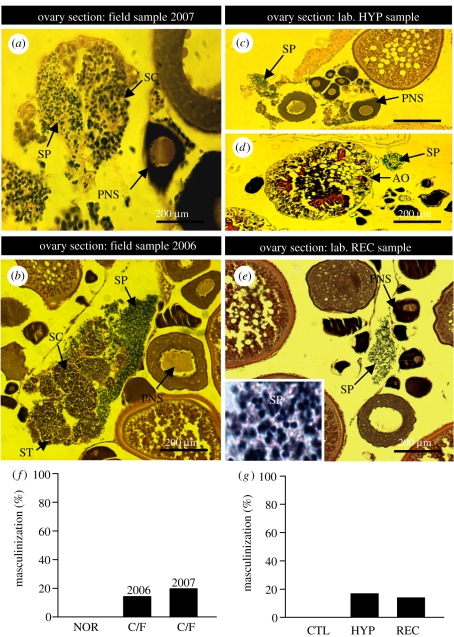
Presence of male germ cells in the ovaries and the percentage of ovarian masculinization in croakers collected at (*a,b,f*) the hypoxic sites (transects C and F) in the nGOM and (*c,d,e,g*) exposed to hypoxia (HYP) in laboratory experiments. AO: atretic oocytes; PNS, peri-nucleolus stage; SC, spermatocytes; ST, spermatids; SP, spermatozoa. NOR, normoxia; CTL, control; REC, recovery.

Histological examination of the ovarian sections of croakers collected from the hypoxic sites revealed that 19.6 per cent (*n* = 71) of them contained groups of male germ cells at several different stages of development, ranging from spermatocytes to fully developed spermatozoa (figure 3*a,f*). Most of these spermatogenic regions contained spermatozoa. Only one or two small spermatogenic regions of male germ cells (diameter: 15–20 µm) were detected in each ovarian section and they were limited to the periphery of the ovary. Spermatogenic cyst-like structures containing male germ cells at the same developmental stage were observed in some ovarian sections, but none of the testicular tissues had well-defined tubular structures characteristic of the normal testis. None of the ovarian sections from fish collected at the reference sites contained any male germ cells (figure 3*f*). Examination of croaker ovaries from a preliminary field study conducted in the nGOM in 2006 [20] revealed that male germ cells were detected in 14.3 per cent of the females collected from the hypoxic sites (*n* = 49), whereas none of the ovarian samples collected from the normoxic site contained spermatogenic tissue (figure 3*b,f*). The specificity of the ovarian masculinization response to hypoxia exposure was evaluated in a controlled laboratory study. Chronic exposure of croakers to low DO for 20 weeks during the period of ovarian crudescence resulted in masculinization, with male germ cells in 16.7 per cent of the ovarian sections, whereas none of the ovarian sections obtained from the normoxic control fish contained any male germ cells (figure 3*c,d,g*). The percentage of ovaries containing male germ cells had not changed five weeks after the fish had been returned to normoxic conditions (figure 3*e,g*).

### (e) Sex ratio

**Figure 4.**
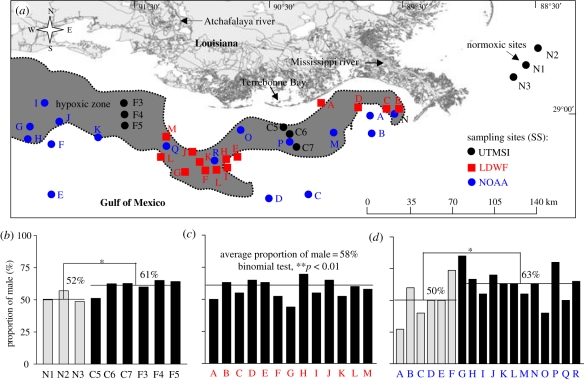
Sex ratio of croakers collected in the normoxic and hypoxic sites in the nGOM during 27 September–11 November 2007. (*a*) Location of sites where fish were collected by the authors (UTMSI, black), by LDWF (blue) and by NOAA (red). Shaded area: extent of hypoxic region. DO: less than 2 mg l^−1^, in July 2007; information obtained from Nancy Rabalais, LUMCON. (*b–d*) Proportion of fish (%) that were males at normoxic (grey) or hypoxic sites (black) collected by (*b*) UTMSI, (*c*) LDWF and (*d*) NOAA. Asterisks denote significant differences between normoxic (reference) and hypoxic sites (nested ANOVA, *p < 0.05, and binomial test, **p < 0.01).

The sex ratio was determined for fish we collected from the six hypoxic and three normoxic sites (UTMSI; black, figure 4*a,b*), for fish collected from 13 hypoxic sites sampled in 8–11 October 2007 by LDWF (blue, figure 4*a,c*), and for fish from 12 hypoxic and six normoxic sites sampled in 10 October–11 November 2007 by NOAA (red, figure 4*a,d*). The combined sex ratio of the fish we collected from the hypoxic sites was 61 per cent males to 39 per cent females—significantly different (*p* < 0.05, *n* = 119) from that at the reference sites (males: 52%, females: 48%; *n* = 61; figure 4*b*). The fish (*n* = 274) collected from the 13 hypoxic sites by LDWF showed a similar sex ratio skewed towards males (58 per cent), which was significantly different from a 50 per cent sex ratio (*p* < 0.01; figure 4*c*). The sex ratios of fish (*n* = 356) collected from the 18 NOAA sites were more variable but showed the same male bias at the hypoxic sites overall, with a mean of 63 per cent males at the hypoxic sites and approximately 50 per cent males at the six sites outside the hypoxic zone (*p* < 0.05; figure 4*d*).

### (f) Reproductive endocrine function

**Figure 5.**
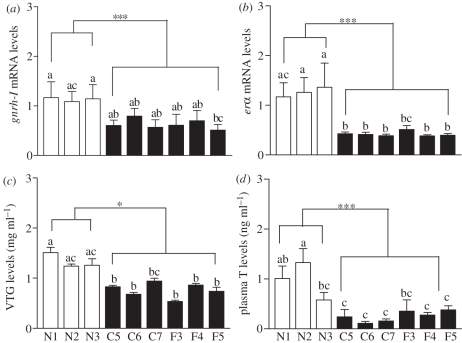
Reproductive endocrine function in female croakers collected from six hypoxic sites (C5–C7, F3–F5) and three normoxic sites (N1–N3) in the nGOM in 27 September–2 October 2007. (*a*) Hypothalamic expression of gonadotrophin-releasing hormone-I (*gnrh-I*) mRNA. (*b*) Hepatic expression of oestrogen receptor alpha (*er*α**) mRNA. (*c,d*) Plasma levels of vitellogenin (VTG) and testosterone (T). Asterisks denote significant differences between normoxic (reference) and hypoxic sites (nested ANOVA, *p < 0.05, ***p < 0.001). Each value represents the mean ± s.e.m. (*n* = 6–13). Individual site differences are indicated with different letters (Fisher's PLSD, *p* < 0.05).

Hypothalamic *gnrh-I* mRNA concentrations, a measure of neuroendocrine function, were significantly reduced compared with reference site controls in fish collected from the C and F transects (figure 5*a*). Hepatic *er*α** mRNA levels, an index of oestrogen signalling, were also significantly reduced in fish from these sites and were less than a third that of the reference controls (figure 5*b*). A similar trend was observed with plasma VTG levels, which were significantly lower in fish collected from the hypoxic sites (figure 5*c*). In addition, plasma T levels, an indication of ovarian steroidogenic capacity, were significantly decreased in fish collected from the hypoxic sites (figure 5*d*).

### (g) Aromatase mRNA expression and activity

**Figure 6.**
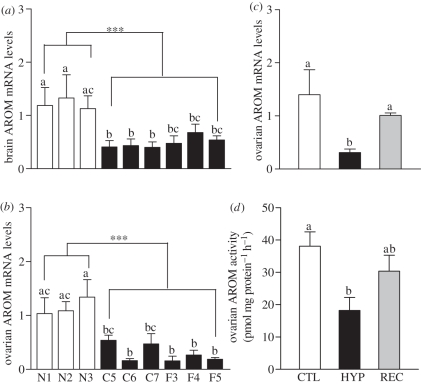
(*a,b*) Effects of exposure to hypoxia in the nGOM and (*c,d*) in laboratory experiments on brain and ovarian (*a,b,c*) aromatase (AROM) mRNA expression and (*d*) enzyme activity in croakers. Asterisks denote significant differences between normoxic (reference) and hypoxic sites (nested ANOVA, ***p < 0.001). Each value represents the mean ± s.e.m. (*n* = 6–12). Individual site (field studies: *a,b*) and treatment differences (laboratory experiments: *c,d*) are indicated with different letters (Fisher's PLSD, *p* < 0.05).

Transcript levels of both brain and ovarian aromatase subtypes were significantly decreased in fish collected from the hypoxic sites compared with those at the reference sites (figure 6*a,b*). Similarly, chronic exposure to hypoxia in the laboratory experiment caused a marked decline in ovarian aromatase mRNA levels (figure 6*c*). Ovarian aromatase activity was also significantly decreased after chronic laboratory exposure to hypoxia (figure 6*d*). Both aromatase mRNA levels and enzyme activities had partially recovered five weeks after the fish had been returned to normoxic conditions and were no longer significantly different from controls (figure 6*c,d*).

## 4. Discussion

The present results provide the first evidence of region-wide impairment of reproductive output in a fish population inhabiting a hypoxic coastal zone. Gonadal growth and gametogenesis were significantly decreased in Atlantic croakers of both sexes collected at the end of September and beginning of October in 2007 from sites approximately 120 km apart that experience chronic seasonal moderate or severe hypoxia compared with fish collected from the reference sites. The production of fully mature eggs and sperm capable of fertilization was less than 20 per cent of that at the reference sites, suggesting that the reproductive output of croakers was severely decreased over a large area of approximately 3000 km^2^ in the nGOM. Recovery of gamete production after this sampling date is highly unlikely because ichthyoplankton surveys indicate that the majority of the croakers in the nGOM spawn in October [21]. A similar impairment of reproductive output was observed in this species exposed to hypoxia in a Florida estuary [7]. However, chronic and repeated decreased reproductive output owing to recurring hypoxia every year over the much larger coastal area investigated in the present study has a much greater potential long-term impact on the size of croaker populations. Atlantic croaker populations have decreased to less than 20 per cent of their historical levels in the nGOM and the croaker commercial fishery has declined dramatically [22], but it is unclear to what extent this decline is due to hypoxia-induced reproductive impairment, because a variety of other contributing factors (such as an increase in bycatch-related mortality) are likely to have major impacts on croaker population abundance in the region [22,23]. Hypoxia-related decreases in the population abundance of fisheries have been reported in a few hypoxic regions such as the Black Sea [24], Baltic Sea [25] and Tokyo Bay [26], but elsewhere seasonal coastal hypoxia has not been associated to date with declines in the population abundance of fishes [5,27]. Nevertheless, the finding that environmental hypoxia exposure severely disrupts the reproduction of a coastal teleost species is a cause for concern because it probably compounds the stress on fish populations in coastal ecosystems associated with other deleterious changes, such as increased fishing pressure, pollution and global warming.

It is not surprising that fish reproduction is susceptible to hypoxia stress, since it is one of the most sensitive stages of an organism's life cycle to stressors, and requires large amounts of energy in excess of that needed for survival [20]. Organisms reduce their energy and ATP use under hypoxic conditions by metabolic suppression in order to compensate for the reduced efficiency and increased energy requirements for anaerobic metabolism, enabling them to survive until normoxic conditions return [6,28]. Most of a fish's available energy is diverted to the production of gametes during the reproductive season. Therefore, a reduction in gonadal growth and gamete production is a highly effective strategy to reduce the energy requirements of fishes during hypoxia exposure and has been observed in iteroparous estuarine fishes such as croakers collected from hypoxic environments [7,13,20,29]. The croaker is a short-lived species [30], so that an individual typically only produces offspring over a few reproductive seasons. Therefore, if large areas of adult fish habitat are subjected to recurring seasonal hypoxia over several years, the reproductive strategy of delaying reproduction may become maladaptive because it could prevent short-lived species such as the croaker from reproducing during their lifetimes, potentially leading to local extinctions of exposed populations.

An unexpected finding of the present study was evidence of gonadal masculinization in approximately 19 per cent of the female fish collected from the hypoxic sites, but not from the reference sites, as indicated by the presence of groups of male germ cells at all stages of development in their ovaries. The specificity of this response was confirmed in the controlled hypoxia laboratory study in which a similar pattern of ovarian masculinization was observed in female croakers. To our knowledge, this is the first report of ovarian masculinization (i.e. the presence of male germ cells) in female fish exposed to hypoxia. The laboratory results indicate that chronic exposure of approximately 7-month-old croakers to hypoxia beginning in June is sufficient to induce ovarian masculinization, consistent with the period when the nGOM coastal region becomes hypoxic and juvenile croakers move into these offshore regions on the continental shelf [14,27]. This sensitivity to environmental induction of intersex at a relatively late developmental stage, presumably after the period of sexual differentiation in croaker, is consistent with reports that the treatment of Nile tilapia with an aromatase inhibitor after the period of gonadal differentiation caused partial ovarian masculinization [31]. These results suggest that continued oestrogen exposure after the period of sexual differentiation is required for normal ovarian development in fish [32]. The presence of spermatogenic tissue in only a proportion of the females in the nGOM is unlikely to be primarily related to differences in their hypoxia exposure histories, because similar results were obtained in the laboratory studies after exposure to a single hypoxia regime. These results instead may indicate differing susceptibilities of a portion of the females to the gonad masculinizing effects of chronic hypoxia exposure.

The marked decrease in expression of aromatase, the key enzyme regulating oestrogen production, is a likely cause of the masculinization of croaker ovaries and the male-biased sex ratio observed in the present study. Aromatase is critical for normal differentiation of the teleost ovary [33], and inhibition of aromatase activity causes ovarian masculinization [32] and can result in the production of phenotypic males from genotypic females [34]. Hypoxia-induced decreases in aromatase activity during gonadal differentiation and at a later gonad-sensitive stage would provide a plausible explanation for the sex ratio being skewed towards males and the presence of testicular tissues in some individuals. Our field results are consistent with laboratory studies in another teleost species, zebrafish, which showed that the sex ratio is skewed towards males and gonadal aromatase activity is inhibited after chronic hypoxia exposure [35]. The decrease in ovarian aromatase activity also probably contributes to the marked decreases in ovarian growth and the production of fully grown oocytes observed in croakers. Oocyte growth is largely dependent on sequestration of VTG, which is synthesized in the liver in response to oestrogen stimulation. The reduction in hepatic *er*α** mRNA expression and circulating levels of VTG indicate oestrogen signalling is impaired in croakers from the hypoxic sites. This decline in oestrogen signalling is probably associated with reduced oestrogen precursor availability in addition to decreased aromatase activity, since plasma T levels were significantly decreased in croakers at the hypoxic sites. Laboratory studies have shown that decreased gonadal steroid production in croakers after hypoxia exposure is due to declines in gonadotrophin secretion, which in turn is associated with downregulation of *gnrh-I* mRNA levels and neuroendocrine function [7]. Therefore, the downregulation of neuroendocrine function in females from the hypoxic sites also probably contributes to the reduced oestrogen signalling, impairment of oocyte growth and gonadal masculinization observed. Aromatase expression in the teleost brain is sexually dimorphic, with twofold higher aromatase activity and mRNA levels in the brains of adult female fish than in males [36,37]. Thus, the marked decrease in brain aromatase mRNA levels in female fish collected from the hypoxic sites suggests that the female brain may also have become masculinized, although the reproductive effects of this are unknown.

There is an urgent need to accurately assess the exposure of ecologically and commercially important pelagic species in the nGOM and other coastal regions to hypoxia and its impacts in order to effectively manage these valuable fishery resources. However, characterizing the exposure of motile species such as croakers to hypoxia in coastal environments is a major technical challenge. Croakers are relatively hypoxia-tolerant demersal (benthopelagic) fishes that feed on benthic organisms and can be captured throughout the summer by trawling in the bottom hypoxic and moderately hypoxic waters (DO: 1.8–3.5 mg l^−1^), but not in anoxic areas [38]. However, the hypoxic waters in the nGOM are confined to the bottom few metres of the water column, so it is unclear whether croakers remain on the bottom or make brief forays into it for feeding. Hypoxia-inducible factors (*hif-1*α** and *hif-2*α**) regulate the many genes involved in adaptation to low oxygen levels and the switch to anaerobic metabolism [39–41]. Croakers collected from currently hypoxic sites in East Bay and the nGOM had elevated tissue expression of *hif-*α*s* [7,20], which indicated that they had been exposed to hypoxic bottom waters over a period of at least 3–7 days, the time required to upregulate *hif-*α*s* in laboratory hypoxia experiments [40]. Thus, although the duration of croaker exposure to hypoxia during a 24 h period in the nGOM hypoxic region remains unknown, the available evidence indicates it is sufficient to induce molecular responses to hypoxia stress, which probably lead to major physiological changes such as metabolic suppression.

Several lines of evidence clearly indicate that the reproductive disruption observed in croakers in the nGOM is primarily associated with chronic hypoxia exposure. The reproductive output of croakers from the reference sites was consistently greater than that from the hypoxic sites over the three sampling seasons conducted to date in 2007, 2008 [29] and 2010 (P. Thomas & M. S. Rahman, unpublished observation). Similar dramatic impairments of ovarian growth, oogenesis and endocrine function have been observed in croakers chronically exposed to hypoxia in controlled laboratory experiments and in hypoxic regions of East Bay [7], as well as in other teleost species [11,13]. In contrast, only moderate inhibition of gonadal growth and oogenesis was observed after exposure of croakers to high sublethal concentrations of Aroclor 1254, benzo[a]pyrene and lead [42]. The pattern of reproductive impairment induced by hypoxia stress in croakers of both sexes, involving masculinization of female gonads, suppression of male and female gametogenesis, inhibition of brain and gonadal aromatase and inhibition of hypothalamic serotonergic function [7], has not been observed to our knowledge with any major environmental contaminant or other physical stressor. It is concluded from these studies that the masculinization and decreased reproductive and endocrine functions observed in croakers collected from hypoxic regions in the nGOM are largely owing to exposure to hypoxia.

The present finding of severe reproductive disruption in an ecologically important fish species throughout a large area in the nGOM hypoxic region has important implications for the management of renewable fishery resources, since it could potentially lead to a gradual decline in the size of croaker populations. In order to restore key components of the ecosystem such as the benthic communities, the goal of the 2001 and 2008 Mississippi River/Gulf of Mexico Watershed Nutrient Task Force Action Plans is to reduce the average size of the hypoxic zone in the nGOM to 5000 km^2^ by reducing nutrient inputs [43]. Although successful implementation of these plans would restore large areas in the nGOM coastal zone to suitable habitats for benthic and demersal benthopelagic organisms, it remains unclear whether it will significantly reduce croaker population hazards owing to other hypoxia effects such as decreased reproductive output. Therefore, it will be necessary to develop population models that predict what size reductions of the nGOM hypoxic zone would protect croaker stocks. Fishery resources in other coastal regions may also become threatened by long-term reductions in reproductive output owing to recurring hypoxia. These effects are not be confined to finfishes, since reproductive impairment was observed in harpactacoid copepods collected from the same hypoxic areas (transects C and F) in the nGOM in 2007 [19]. Human activities are projected to have major impacts this century on our planet, especially on coastal regions. In addition to increases in temperature and sea levels, another major global change, the increased extent of seasonal hypoxia, now extending to 250 000 km^2^ worldwide, could dramatically affect fishery resources in coastal regions and the livelihoods of people that depend upon them. Therefore, reproductive output of fish inhabiting other coastal regions experiencing recurring seasonal hypoxia should be investigated for evidence of hypoxia-related declines.
